# LIP1 Regulates the Plant Circadian Oscillator by Modulating the Function of the Clock Component GIGANTEA

**DOI:** 10.3390/cells13171503

**Published:** 2024-09-08

**Authors:** Anita Hajdu, Dóra Nyári, Kata Terecskei, Péter Gyula, Éva Ádám, Orsolya Dobos, Zsuzsanna Mérai, László Kozma-Bognár

**Affiliations:** 1 Department of Genetics, Faculty of Sciences and Informatics, University of Szeged, H-6726 Szeged, Hungary; hajdu.anita@brc.hu (A.H.); nyari.dora@brc.hu (D.N.); 2 Institute of Plant Biology, HUN-REN Biological Research Centre, H-6726 Szeged, Hungary; kata1213@gmail.com (K.T.); dr.eva.adam@gmail.com (É.Á.); dobos.orsolya.katalin@gmail.com (O.D.); 3 Department of Medical Genetics, Faculty of Medicine, University of Szeged, H-6720 Szeged, Hungary; 4 Doctoral School in Biology, Faculty of Science and Informatics, University of Szeged, H-6726 Szeged, Hungary; 5 Institute of Genetics and Biotechnology, Hungarian University of Agriculture and Life Sciences, H-2100 Gödöllő, Hungary; gyula.peter@uni-mate.hu; 6 Gregor Mendel Institute of Molecular Plant Biology GmbH, 1030 Vienna, Austria; zsuzsanna.merai@gmi.oeaw.ac.at

**Keywords:** arabidopsis, circadian clock, GIGANTEA, small GTPase LIP1

## Abstract

Circadian clocks are biochemical timers regulating many physiological and molecular processes according to the day/night cycles. The function of the oscillator relies on negative transcriptional/translational feedback loops operated by the so-called clock genes and the encoded clock proteins. Previously, we identified the small GTPase LIGHT INSENSITIVE PERIOD 1 (LIP1) as a circadian-clock-associated protein that regulates light input to the clock in the model plant *Arabidopsis thaliana*. We showed that LIP1 is also required for suppressing red and blue light-mediated photomorphogenesis, pavement cell shape determination and tolerance to salt stress. Here, we demonstrate that LIP1 is present in a complex of clock proteins GIGANTEA (GI), ZEITLUPE (ZTL) and TIMING OF CAB 1 (TOC1). LIP1 participates in this complex via GUANINE EX-CHANGE FACTOR 7. Analysis of genetic interactions proved that LIP1 affects the oscillator via modulating the function of GI. We show that LIP1 and GI independently and additively regulate photomorphogenesis and salt stress responses, whereas controlling cell shape and photoperiodic flowering are not shared functions of LIP1 and GI. Collectively, our results suggest that LIP1 affects a specific function of GI, possibly by altering binding of GI to downstream signalling components.

## 1. Introduction

Circadian rhythmicity provides time-of-day specific regulation for a wide range of processes in living organisms. Driven by this regulation, processes from gene expression to locomotor activity are scheduled to the most appropriate hours of the day, but are tuned down when they are needless. This results in saving resources and energy, which advantage probably promoted the evolution of circadian clocks, the biological timers that create and maintain circadian rhythms [1].

In eukaryotes, circadian clocks are built of clock genes and corresponding clock proteins that regulate the expression of each other, forming transcriptional/translational feedback loops [2]. The primary app. 24 h oscillation is generated at the level of clock gene transcription and is transduced to rhythmically modulate the expression of a significant number of genes. The time-specific orchestration of gene expression eventually leads to the rhythmic regulation of biochemical, physiological or behavioural processes. Daily regulation is useful for the host only if it corresponds to the real time of the environment. This requires that the oscillator keeps synchrony with the outer light/dark cycles via a process called entrainment, during which periodic environmental signals (e.g., light and temperature) set the phase of the clock. Since the oscillator receives signals from nearly all photoreceptors covering the visible part of the spectrum, all wavelengths of light can contribute to the entrainment of the plant clock, albeit with different efficiency [3]. The mechanism operating the oscillator (i.e., negative feedback loops) is highly conserved in eukaryotes, whereas the actual components are structurally divergent in the kingdoms of animals, fungi and plants [4].

In the model plant *Arabidopsis thaliana*, the first identified oscillator components were the transcription factor CIRCADIAN CLOCK ASSOCIATED 1 (CCA1) and its homolog LATE ELONGATED HYPOCOTYL (LHY), and the pseudo response regulator TIMING OF CAB EXPRESSION 1 (TOC1) [5,6]. CCA1/LHY are expressed in the morning and repress *TOC*1 transcription during the day via direct binding to the *TOC*1 promoter. In turn, evening-expressed TOC1 represses *CCA1*/*LHY* transcription in the evening and at early night [7]. TOC1, also called PSEUDO-RESPONSE REGULATOR 1 (PRR1), is the member of a small gene/protein family. *PRR*9, *PRR*7, *PRR*5, *PRR*3 and *TOC*1/*PRR*1 are expressed sequentially, showing peaks of transcription from the morning (*PRR9*) until dusk (*TOC*1/*PRR*1) [8]. These transcriptional repressors inhibit the transcription of *CCA1*/*LHY* during the second half of the day and the first half of the night [9]. The evening-expressed EARLY FLOWERING 3 (ELF3), EARLY FLOWERING 4 (ELF4) and LUX ARRHYTHMO (LUX) proteins form the tripartite evening complex (EC), which represses the *PRR* genes during the second half of night, thus enabling the activation of *CCA1*/*LHY* in the morning [10]. A complex formed by LIGHT-REGULATED WD1 (LWD1), TEOSINTE BRANCHED 1-CYCLOIDEA-PCF20 (TCP20) and TCP22 proteins plays an important role in the promotion of *CCA1* transcription around dawn [11]. Components of the EC are also repressed by CCA1/LHY, ensuring that the indirect positive effect of the EC on *CCA*1/*LHY* follows the inhibition provided by the PRR family. Among the few positively acting components of the plant clock, NIGHT LIGHT-INDUCIBLE AND CLOCK-REGULATED GENE (LNK) and REVEILLE 8 (RVE8) form a transcriptionally active protein complex that promotes the expression of *PRR5* and *TOC1* [12].

Although the highly complex genetic network that underlies the plant circadian oscillator is primary operated by transcriptional regulation of the clock genes, the timely degradation of the corresponding clock proteins is essential to maintain the 24 h oscillation. Two PRR proteins, PRR5 and TOC1, are ubiquitinated and subsequently degraded by the 26S proteasome. The F-box protein ZEITLUPE (ZTL) plays an essential adaptor function connecting the PRR5 and TOC1 proteins with the ubiquitin ligase complex mediating (poly)ubiquitination [13,14]. Although *ZTL* transcription is not rhythmic, ZTL protein level shows a clear peak around dusk, which is the result of the stabilization effect of GIGANTEA (GI) [15]. GI is an evening-expressed oscillator component, which simultaneously binds to the UBIQUITIN-SPECIFIC PROTEASE 12/13 (UBP12/13) and the ZTL proteins. UBP12/13 do not interact with ZTL directly, but being recruited by GI, these proteases deubiquitinate ZTL and thus promote its accumulation around dusk [16].

GI is a relatively large 127.9 kDa protein without any known domain structure or biochemical function. In addition to its role in the circadian oscillator, GI has been implicated in the regulation of flowering time, photomorphogenesis, in the adaptation of various adverse environmental conditions, such as low or high temperatures, high salinity and water deficiency, and in defence responses to biotic stresses, such as fungal infections [17,18]. It has been suggested that the exceptionally diverse functions of the protein could be mediated via physical interactions of GI with diverse signalling proteins specifically acting in various regulatory pathways [19]. For example, in long day conditions, GI and FLAVIN-BINDING, KELCH REPEAT, F BOX 1 (FKF1) proteins are co-expressed and assemble in a complex promoting the degradation of CYCLING DOF FACTORs (CDFs) [20]. The elimination of CDFs relieves transcriptional repression of *CONSTANS* (*CO*), resulting in the accumulation of the CO protein, which activates the transcription of *FLOWERING LOCUS T* (*FT*) and thus initiates flowering [21]. Interestingly, a missense allele of *GI* (*gi-611*) affects the speed of the circadian clock, but does not show the characteristic late-flowering phenotype of *gi* mutants [22]. The fact that different functions of GI can be separated by amino acid changes at different parts of the protein supports the hypothesis that multifaceted roles of GI could be explained by diverse protein–protein interactions.

LIGHT INSENSITIVE PERIOD (LIP1) is the first small GTPase that has been functionally linked to the plant circadian clock [23]. The null alleles of LIP1 (*lip1-1*, *lip1-2*) display a short period phenotype, but the molecular mechanism by which LIP1 affects the oscillator remain elusive. Here, we provide molecular and genetic evidence that LIP1 modulates the pace of the clock by indirectly binding to GI via the GUANINE EXCHANGE FACTOR 7 (GEF7) protein. Previously, we showed that, independent of its circadian function, LIP1 affects photomorphogenesis and salt-stress tolerance via unknown mechanisms [24]. In this work, we demonstrate that although both LIP1 and GI regulate these processes, they act independently. LIP1 plays a role in controlling the shape of epidermal pavement cells, most likely via the regulation of endoreplication in a light-dependent manner [24]. Here, we demonstrate that this function of LIP1 is not shared with GI. Moreover, we show that LIP1 does not modulate the function of GI in the regulation of flowering time. Taken together, our results suggest that LIP1 may alter the interaction of GI with partners implicated in clock regulation, but does not affect the binding of GI to downstream signalling factors that target light regulation, salt stress tolerance or flowering time determination.

## 2. Materials and Methods

### 2.1. Plant Materials and Growth Conditions

The Columbia-0 (Col-0) accession of Arabidopsis (*Arabidopsis thaliana*) was used as the background for all the experimental lines. The mutant lines used here have been described earlier: *lip1-2* [23], *toc1-4* [25,26], *ztl-3* [27], *cca1-1* [28] (Ws background back-crossed with Col-0 five times). The *gi-101* allele was identified from the SALK collection (SALK_092757) and ordered from the Eurasian Arabidopsis Stock Centre (uNASC), id: N592757. PCR primers were designed using the T-DNA Primer Design Tool (http://signal.salk.edu/tdnaprimers.2.html (accessed on 11 May 2021.)) and used for allele-specific genotyping. Primer sequences are shown in Supplementary Table S1. The *CCR2:LUC* marker was introgressed from a transgenic Col-0 line [29] to *lip1-2.* The resulting *lip1-2 CCR2:LUC* line was crossed to *ztl-3*, *cca1-1* and *gi-101*. The wild type, the single and the double mutants were all selected from the corresponding F2 segregating populations. The *toc-4* line carried a *CAB2:LUC* reporter, which was introgressed in *lip1-2* by crossing. Again, the wild type, the two single mutants and the double mutant were selected from the F2 population. This protocol ensured the homogenous genetic background of the lines (except for the *lip1-2* and the clock gene mutations, of course) that had to be assessed for clock function. 

Seeds were surface-sterilized, stratified at 4 °C for 3 days and then grown in 12 h fluorescent white light (75 µmol m^−2^ s^−1^ fluence rate)/12 h dark cycles at 22 °C for 7 days, unless indicated otherwise. Seedlings for cell morphology determinations were grown on half-strength Murashige and Skoog (MS) media supplemented with 1% (*w/v*) sucrose (MS1). Plants for RNA isolation, luminescence detection and salt tolerance tests were grown on MS media supplemented with 3% (*w/v*) sucrose (MS3). For hypocotyl elongation tests, seedlings were sown on wet filter paper. Special growth conditions are described below or in the corresponding figure legends.

### 2.2. Analysis of Gene Expression

For RNA isolation and mRNA quantification, plants were grown in 12 h light/12 h dark photocycles for 7 days and then transferred to continuous monochromatic red light (cR) at 5 µmol m^−2^ s^−1^ fluence rate. Samples were harvested in 3 h intervals for 3.5 days, started 24 h after the transfer to cR. Total RNA was isolated with the Nucleospin RNA Plant kit (Macherey-Nagel) according to the manufacturer’s recommendations. An amount of 1 µg RNA was used in first strand synthesis reactions using the RevertAid First Strand cDNA Synthesis Kit with random hexamer oligonucleotides (Thermo Scientific) according to the manufacturer’s suggestions. The final reaction volume of 20 µL was diluted to 100 µL with nuclease-free water and used as template in in qPCR assays. A typical qPCR reaction mixture of 15 µL volume contained 2.5 µL of the diluted cDNA sample, 7.5 µL 2× qPCRBIO SyGreen Mix Hi-ROX master mix (PCR Biosystems), and primers (Merck/Sigma-Aldrich) for amplification at a final concentration of 300 nM each. Assays were run on an ABI Prism 7300 real-time PCR system according to the recommendations of the manufacturer of the SYBR Green master mix. Each sample was assayed in triplicate within a single experiment, and experiments were replicated three times using biologically independent sample sets. Calculations were performed according to the standard curve method. Expression values were normalised to the values obtained for the *TUBULIN 2* and *TUBULIN 3* (*TUBULIN 2/3*, *TUB*) house-keeping genes.

The in vivo luminescence measurements were taken essentially as described [30] Briefly: wild-type and different single or double mutant seedlings expressing the *CCR2:LUC* or the *CAB2:LUC* marker were grown in 12 h white light/12 h dark cycles for 6 days and then individually transferred to the wells of 96-well microplates (OPTIPLATE-96 F, PerkinElmer, Waltham, Massachusetts, United States) containing 250 µL MS3 medium. Then, 25 µL of 2.5 mM D-luciferin (Biosynth) was pipetted to each seedling. Plates were transferred to the TopCount NXT automated luminometer at dawn (the time of dark-to-light transition) in continuous red light (5 µmol m^−2^ s^−1^) conditions. Luminescence was monitored for 4–5 days; individual seedlings were measured hourly. The counts collected during the assay were processed in Microsoft Excel to produce graphs. Time series data were evaluated using the Biological Rhythms Analysis Software System v2.1.3. (BRASS2) software package (was downloadable from http://www.amillar.org (accessed on 17 October 2006), now discontinued and replaced by the online platform of Biological Data Repository, BioDare at https://www.biodare.ed.ac.uk (accessed on 26 February 2020)) running Fast Fourier Transform Non Linear Least Squares (FFT-NLLS) analysis to estimate periods in the 15–35 h circadian range.

### 2.3. Yeast Two-Hybrid Screening and Testing Pairwise Protein Interactions

To screen for proteins interacting with LIP1, a normalized Arabidopsis cDNA library cloned in pGADT7 vector and transformed into yeast strain Y187 (MATα) was used (Mate & Plate™ Library—Universal Arabidopsis, Clontech/Takara). The coding region of *LIP1* was cloned in pGBKT7 plasmid and transformed into yeast strain PJ69-4A (MATa). The strains were mixed and incubated at 30 °C for 24 h for mating and then plated on Ade-/Leu-/Trp- Synthetic Defined (SD) (ALW/SD) medium and incubated at 30 °C for 5 days. Growing colonies were diluted in sterile water and plated on ALW/SD and His-/Leu-/Trp- SD (HLW/SD) medium supplemented with 10 mM 3-Amino-1,2,4-triazole (3-AT) in order to select strains representing the strongest interactions. The pGADT7 plasmid carrying the gene of the interacting protein was isolated from the selected strains, amplified in *E*. *coli* XL-1 Blue cells and then co-transformed with pGBKT7 *LIP1* into PJ69-4A to verify the interaction. In the next step, the inserted DNA fragment was sequenced from the verified pGADT7 clones, and the corresponding genes were identified. All the clones contained truncated derivatives of different genes. The full-length versions of these were cloned in pGADT7 to re-test interaction with LIP1.

To test pairwise protein interactions, the full-length cDNA fragments of *LIP1*, *GEF7*, *GI*, *ZTL* and *TOC1* were cloned in both pGADT7 and pGBKT7 vectors (Clontech) in frame with the GAL4 transcriptional activator domain or with the GAL4 DNA-binding domain, respectively. The various pGADT7-pGBKT7 pairs were co-transformed in PJ69-4A cells. Transformed cells were plated on Leu-/Trp- (LW) SD and Ade-/Leu-/Trp- (ALW) SD agar plates and were grown at 30 °C for 5 days. For β-galactosidase enzyme activity assay, three independent colonies were picked up from LW SD or ALW SD plates and inoculated into LW SD liquid media, and were shaken at 30 °C until density reached OD600 = 0.8, when the assay (using O-nitrophenyl-β-D-galactopyranoside as substrate) was carried out [31].

### 2.4. Analysis of Pavement Cell Morphology

Seedlings were grown in 12 h light/12 h dark cycles for 8 days and then cleared overnight in a solution of 160 g of chloral hydrate, 100 mL of water, and 50 mL of glycerol. After clearing, cotyledons were placed in between a glass slide and a cover slip, and pavement cells were observed with an Olympus Bx51 microscope equipped with Differential Interference Contrast (DIC) optics (Olympus, Tokyo, Japan). Images were captured on an Olympus DP72 digital camera. The area and perimeter of pavement cells were determined with Metamorph software (version 7.1). Shape factor was calculated as 4π area perimeter^−2^. 

### 2.5. Physiological Assays

For qualitative salt tolerance assays, seedlings were germinated and grown in 12 h light/12 h dark cycles for 14 days on MS3 media with or without 100 mM NaCl. To document the developmental state of the different genotypes, plates were turned upside down and scanned. For the quantitative assessment of the effect of high salinity on germination rate, seeds were sown on media with or without 200 mM NaCl. After stratification, plates were transferred to 12 h light/12 h dark conditions and were investigated using a stereomicroscope daily for 5 days. Seeds with clearly emerging radicles were considered germinating and were counted. Germination rate was calculated by dividing the number of germinating seeds by the total number of seeds on the plate. The experiment was replicated three times.

To measure the inhibitory effect of light on hypocotyl elongation, dry seeds were sown on 3 layers of wet filter paper in 9 cm Petri dishes and stratified in the dark at 4 °C for 3 days. Germination was induced and synchronised by white light illumination (75 µmol m^−2^ s^−1^) for 8 h. Plates were then incubated in the dark at 22 °C for 16 h and then transferred to continuous monochromatic red (20 µmol m^−2^ s^−1^), blue (2 µmol m^−2^ s^−1^) or far-red (1 µmol m^−2^ s^−1^) light, or were kept in the dark. After 4 days of growth, hypocotyls of 30–40 seedlings per genotype per light condition were measured and normalized to the length of the dark-grown seedlings. Measurements were repeated four times.

To determine flowering time of the different genotypes, seeds were sown on soil, stratified in the dark at 4 °C for 7 days and then transferred to 22 °C under short day (SD, 8 white light/16 h dark) or long day (LD, 16 h white light/8 h dark) conditions. Flowering time was recorded as the number of rosette leaves at the time of bolting. A total of 12–15 plants were analysed per genotype per condition. The full set of assays (i.e., SD + LD) was repeated three times.

### 2.6. Statistical Analysis

Statistical significance was evaluated with *t*-tests or Duncan’s tests, according to the nature of numerical data. Calculations were performed with SigmaStat^®^ 4.0 software.

## 3. Results

### 3.1. Pattern and Level of Clock Gene Expression in the lip1-2 Mutant

**Figure 1:**
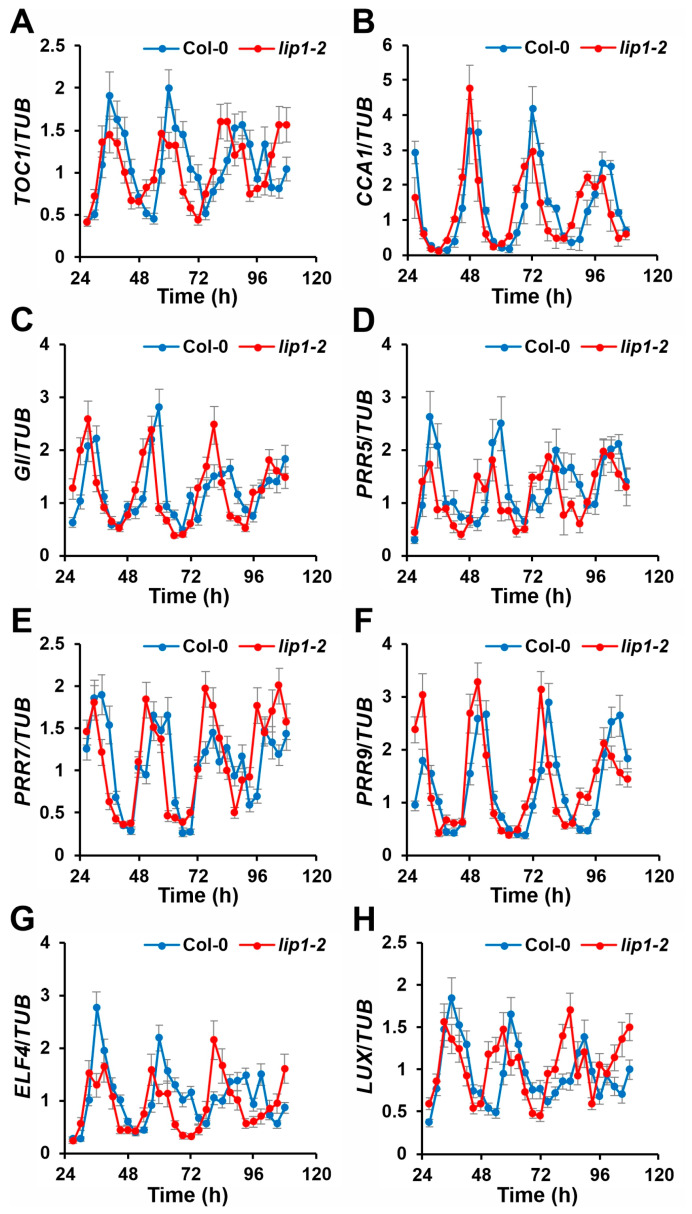
mRNA accumulation pattern of clock genes in the *lip1-2* mutant. Wild-type (Col-0) and *lip1-2* mutant seedlings were grown in 12 h light/12 h dark photocycles for 7 days and transferred to continuous red light (5 µmol m^−2^ s^−1^). Samples were harvested at 3 h intervals, starting 27 h after the transfer. mRNA levels of *TOC1* (**A**), *CCA1* (**B**), *GI* (**C**), *PRR5* (**D**), *PRR7* (**E**), *PRR9* (**F**), *ELF4* (**G**), and *LUX* (**H**) were determined by qPCR and normalised to the corresponding *TUBULIN 2/3* (*TUB*) mRNA levels. Average values of 3 independent replicates are plotted, error bars represent standard error values.

We have demonstrated previously that LIP1 affects the circadian clock by mediating light signalling to the oscillator [23]. Input light signals may affect the level, the activity or subcellular localization of oscillator components to set the pace and phase of the circadian clock. In order to test the effect of LIP1 on clock gene expression, Col-0 wild-type and *lip1-2* mutant seedlings were entrained to 12 h light/12 h dark photocycles for a week and then transferred to continuous red light at relatively low fluence rate (5 µmol m^−2^ s^−1^), where the short period phenotype of *lip1-2* mutants is readily detectable [23,24]. The accumulation pattern and level of selected clock gene mRNA molecules were analysed by qPCR assays. The genes were selected to represent the different regulatory loops, but also to include the first identified main components (*CCA1*, *TOC1*), genes with sequential peak times during the day (*PRR5*, *7*, *9*) and key elements of the evening complex (*LUX*, *ELF4*) as well. Figure 1 shows that rhythmic mRNA accumulation of all tested genes displayed shorter periods in the *lip1-2* mutant compared with the wild type control. However, mRNA levels did not change consistently in the *lip1-2* mutant in either case. These data suggested that altered level of clock gene expression probably does not underlie the periodic phenotype of *lip1-2*; thus, the primary and direct effect of LIP1 on the oscillator is not the transcriptional regulation of clock genes.

### 3.2. Identification of Proteins Interacting with LIP1

**Figure 2:**
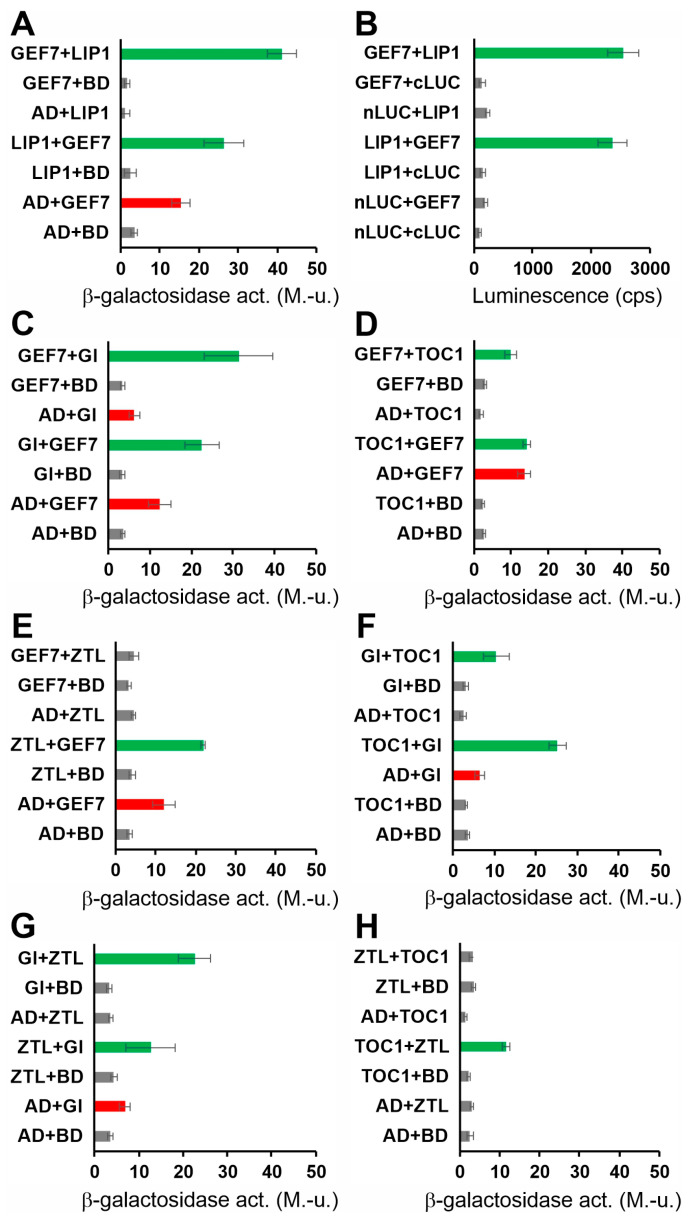
Identification of proteins directly or indirectly interacting with LIP1. Full-length LIP1, GEF7, GI, TOC1 and ZTL proteins fused to the transcriptional activation domain (AD) or the DNA-binding domain (BD) of the GAL4 transcription factor were co-expressed in yeast (PJ69-4A) cells (**A**,**C**–**H**). Pairwise interactions were tested in either (AD and BD) configurations. For each combination, the first or the second indicated protein carried the AD or the BD fusion tag, respectively. AD and BD correspond to controls, where these GAL4 derivatives were expressed without foreign fusion partners. β-galactosidase activity, reporting the activation of the *lacZ* marker and therefore the strength of interaction between the two given fusion proteins, was determined from liquid-cultured transformant cells. Green bars indicate activation above the background levels (grey bars). GEF7 and GI fused to BD were able to activate the marker gene without any interacting partners (transactivation, red bars). The assays were repeated 3–4 times with essentially the same results. Error bars represent standard error values of 3 technical repeats of a representative assay. M.-u.: Miller-units. The interaction between LIP1 and GEF7 was also tested by luciferase complementation assays (**B**). LIP1 or GEF7 fused to the N- or C-terminal fragment of firefly luciferase (nLUC or cLUC) in either configuration were coexpressed in *E. coli* BL21 Rosetta cells along with the corresponding controls. Luminesce of freshly grown bacterial cultures was detected in a luminometer. Error bars represent the standard error values of 3 independent assays. cps: counts per second.

Since the results above suggested that LIP1 may exerts its clock-related function at posttranscriptional/protein level, we aimed at identifying proteins thorough which this regulation could take place. First, we tested the interactions between LIP1 and several clock proteins (CCA1, TOC1, GI, ZTL, ELF4, ELF3, LUX, PRR9) using the GAL4-based yeast two-hybrid (Y2H) system without any positive results. To expand the range of potential partners, in the next step, we performed a yeast two-hybrid (Y2H) screen employing LIP1 as bait. We isolated seven clones encoding protein fragments that interacted with LIP1 in a reproducible manner. However, only one of these retained the ability for interaction when the corresponding full-length protein was co-expressed with LIP1 (Figure 2A). The gene is designated as AT5G02010 and encodes for ROP (RHO OF PLANTS) GUANINE NUCLEOTIDE EXCHANGE FACTOR 7 (ROPGEF7, GEF7 hereafter in the text). GEF7 belongs to the family of ROPGEF proteins consisting of 14 members in Arabidopsis [32,33]. These proteins facilitate the replacement of GDP by GTP bound to the plant-specific Rop GTPases, leading to the activation of these signalling factors [32]. The LIP1-GEF7 interaction was verified by a Luciferase Complementation Assay (Figure 2B) in *E. coli* cells, overcoming the problem of transactivation by GEF7 in the Y2H system. This result also suggested that no plant-specific posttranslational modifications are required for the establishment of LIP1–GEF7 interaction. To reveal potential links to the oscillator, we tested interactions between GEF7 and clock proteins CCA1, TOC1, GI, ZTL, ELF4, ELF3, LUX and PRR9. Significant binding to GEF7 was detected in the case of GI, TOC1 and ZTL (Figure 2C–E). Interestingly, it has been demonstrated previously that ZTL promotes the degradation of TOC1 [34], whereas GI stabilizes ZTL in a light-dependent manner by recruiting UBIQUITIN-SPECIFIC PROTESASE 12 and 13 to the ZTL-TOC1 complex [15,16]. We verified the GI-ZTL and the ZTL-TOC1 interactions in the Y2H system (Figure 2G,H), but also demonstrated a physical association between TOC1 and GI (Figure 2F) that has not been reported before. These results suggest that GEF7 may have a dual function: acting as a guanine exchange factor to promote the GDP/GTP exchange for LIP1, and serving as an adaptor to bring LIP1 and certain clock proteins into close proximity. However, further experiments are needed to determine the functional significance of the protein interaction patterns reported here.

### 3.3. Genetic Analysis Identifies GI as the Clock Component Targeted by LIP1

**Figure 3:**
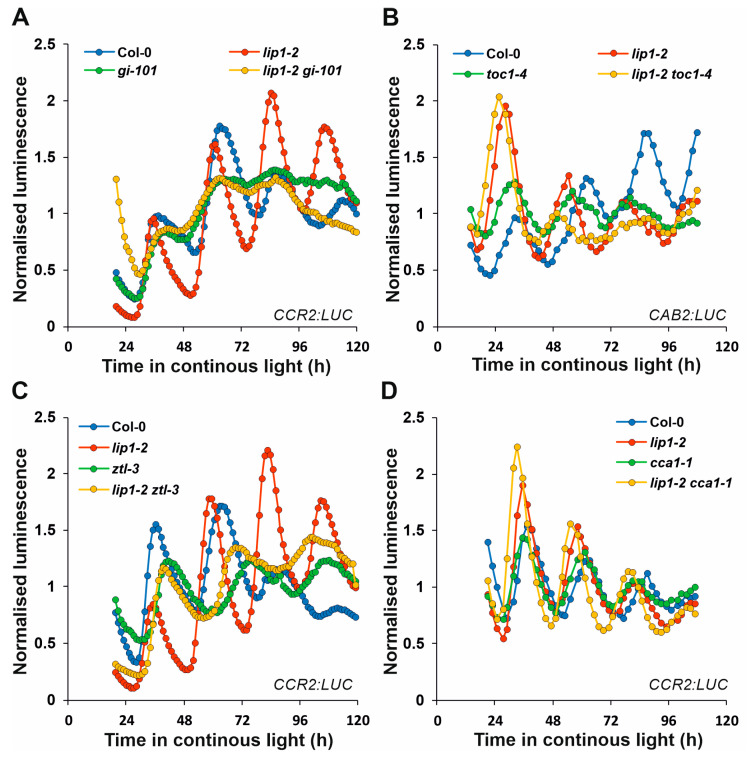
LIP1 requires GI to affect the circadian clock. Seedlings of the indicated genotypes carrying *CCR2:LUC* (**A**,**C**,**D**) or *CAB2:LUC* (**B**) reporter genes were grown in 12 h light/12 h dark photoperiods for 7 days and transferred to continuous red light (5 µmol m^−2^ s^−1^), where luminescence was monitored. For each individual seedlings, values were normalised to the average of all counts collected during the course of the assay. The means of normalised data from 24 seedlings for each genotype are plotted. Experiments were repeated 3 or 4 times.

In order to reveal the functional consequences of the indirect interactions described above and to test if one of the complex-forming clock proteins represents the entry point of LIP1-derived in the oscillator, double mutants were generated by crossing *lip1-2* to *gi-101*, *toc1-4*, *ztl-3* or *cca1-1*. The *cca1-1* mutant was used as control, since neither direct nor indirect interaction was detected between CCA1 and LIP1. The mutant combinations carried the *CCR2:LUC* or the *CAB2:LUC* reporters facilitating the analysis of the circadian phenotypes. Plants, including the wild-type and the single mutant controls, were assayed in low-intensity red light (Figure 3). Visual inspection of rhythmic traces indicated additive period phenotypes for *lip1-2* and *toc1-4* (Figure 3B), *ztl-3* (Figure 3C) and *cca1-1* (Figure 3D). Estimates of free-running periods verified this observation with quantitative data (Table 1). The period of *lip1-2 ztl-3* was in between the two parent singles, whereas the periods of *lip1-2 toc1-4* and *lip1-2 cca1-1* were significantly shorter compared with the parental lines. In contrast, *lip1-2 gi-101* produced *CCR2:LUC* rhythms with periods indistinguishable from that of *gi-101*, but significantly longer than that of the *lip1-2* single (Figure 3A, Table 1). Moreover, the reduction in amplitude seen in *gi-101* was also clearly observable in *lip1-2 gi-101*. These data demonstrate that GI is epistatic to LIP1 in the regulation of the circadian oscillator.

### 3.4. Additive Effects of LIP1 and GI on Salt Stress Responses and Photomorphogenesis

In addition to its function in the regulation of the circadian clock, LIP1 was shown to control responses to salt stress, light-dependent hypocotyl elongation and endoreplication [24]. Since we showed that LIP1 affects the clock through GI, we aimed at testing if other phenotypes of the *lip1-2* mutants also depend on GI.

**Figure 4:**
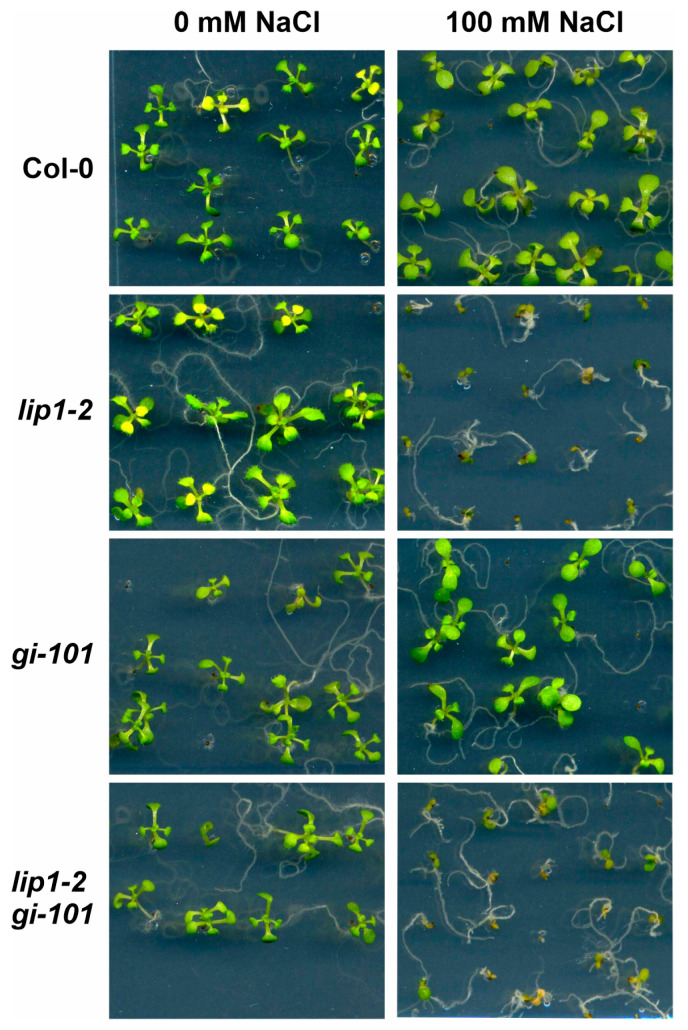
Impaired development of *lip1-2* and *lip1-2 gi-101* mutants in high salinity conditions. Col-0, *lip1-2*, *gi-101* and *lip1-2 gi-101* seedlings were grown in 12 h light/12 h dark photocycles for 14 days on media with or without 100 mM NaCl.

**Figure 5:**
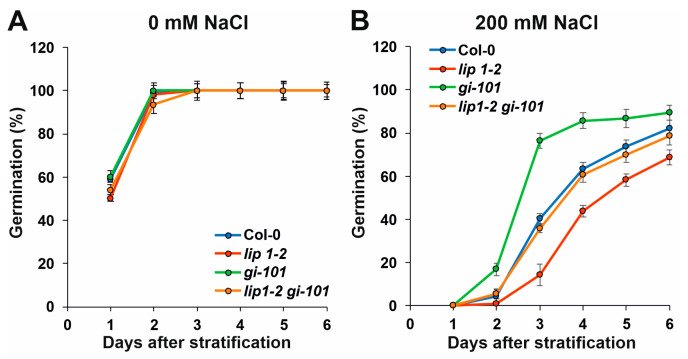
Additive effects of *lip1-2* and *gi-101* mutations on salt stress responses of germination. Col-0, *lip1-2*, *gi-101* and *lip1-2 gi-101* seedlings were grown in 12 h light/12 h dark photocycles on media with (**B**) or without (**A**) 200 mM NaCl. The number of seedlings with emerged radicles were counted daily and expressed as the percentage of the total number of seeds. Error bars represent standard error (SE) values, n = 48−72.

LIP1 is required for efficient tolerance of high salinity, as the development of *lip1-1* and *lip1-2* mutants was severely impaired at 100 mM NaCl, which was clearly tolerated by WT plants (Figure 4) [24]. GI is a negative regulator of the SALT OVERLY SENSITIVE (SOS) pathway that acts as the first defence line in high-salinity conditions. Salt stress triggers interaction of the SOS2 kinase and the SOS3 activating protein to form the functional SOS2-SOS3 kinase complex that phosphorylates and activates the plasma membrane localized SOS1 Na^+^/H^+^ antiporter pumping out the excess Na+ from the cells [35]. Under normal conditions, GI attenuates the activity of the SOS pathway by binding and sequestering SOS2. Upon salt stress, GI is targeted to degradation, SOS2 is released and SOS1 is activated [36,37,38]. Accordingly, *gi* mutants show enhanced tolerance to salt stress, though this was not apparent in our growth assay (Figure 4). The *lip1-2 gi-101* double mutant behaved like *lip1-2*, which could indicate at first glance that LIP1 functions downstream of GI in controlling salt-stress responses (Figure 4). In order to clarify the genetic/functional interaction of *lip1-2* and *gi-101* mutations, seeds were sown on control and 200 mM NaCl media, and germination rate was counted daily (Figure 5A and Figure 5B, respectively). This quantitative assay enabled the measurement of contribution of the single and combined mutation to the response, which could be masked in the qualitative growth assay (Figure 4). Indeed, the higher tolerance of *gi-101* and the hypersensitivity of *lip1-2* plants were clearly demonstrated in Figure 5B. Importantly, the response of *lip1-2 gi-101* was in between the single mutants and was very similar to that of the wild-type plants. The germination capacity of the seeds used in the assay was very similar (Figure 5A), indicating that the differences obtained on 200 mM NaCl are due to the different sensitivity of the mutants to high salinity. Taken together, the results demonstrated that LIP1 and GI function independently in the regulation of salt stress.

**Figure 6:**
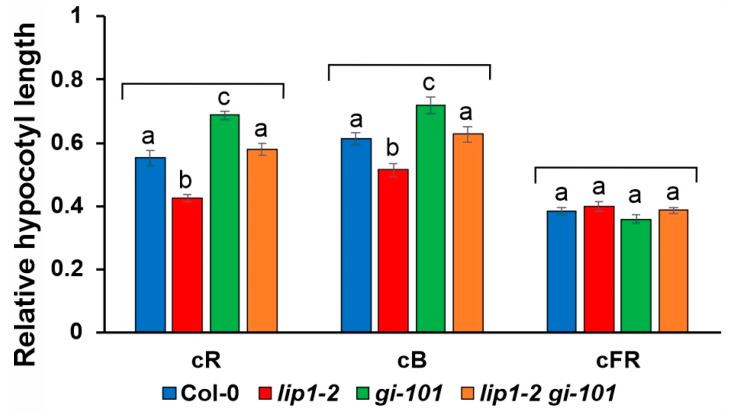
LIP1 affects photomorphogenic responses independently of GI. Wild-type Col-0, *lip1-2*, *gi-101* and *lip1-2 gi-101* mutant seedlings were grown in continuous red (cR, 20 µmol m^−2^ s^−1^), blue (cB, 2 µmol m^−2^ s^−1^) or far-red (cFR, 1 µmol m^−2^ s^−1^) light for 4 days. Hypocotyl lengths were measured and normalised to the corresponding dark-grown hypocotyl lengths. A total of 30–40 seedlings were analysed for each genotype and light condition. Error bars indicate standard error (SE), and different letters show significant differences at *p* < 0.05 (Duncan’s test).

Both LIP1 and GI play a role in light-controlled hypocotyl elongation, although they exert opposite effects: compared with wild-type plants, loss of function alleles of *LIP1* or *GI* produce shorter or longer hypocotyls, respectively, when grown in continuous red or blue light [23,24,39]. However, none of them are involved in signal transduction mediated by continuous far-red light [24,40]. To test genetic interaction between LIP1 and GI for these phenotypes, hypocotyl lengths of seedlings of different genotypes were determined after 4 days of growth in continuous red, blue and far-red light and in darkness. In order to reflect the light-controlled component of hypocotyl elongation, hypocotyl length of light-grown seedlings was normalized to the height of the corresponding dark-grown plants. Figure 6 shows that regarding the inhibition of hypocotyl elongation by red and blue light *lip1-2* was hypersensitive, whereas *gi-101* was hyposensitive, as reported earlier. The *lip1-2 gi-101* double produced hypocotyl lengths intermediate between the two single mutants and mimicked the wild type. As expected, none of the mutants showed alterations from the wild type in far-red light. The additivity of the light-dependent phenotypes demonstrates that LIP1 and GI affect photomorphogenesis via different molecular routes.

### 3.5. Regulation of Cell Morphology and Control of Flowering Time Are Not Shared Functions of LIP1 and GI

**Figure 7:**
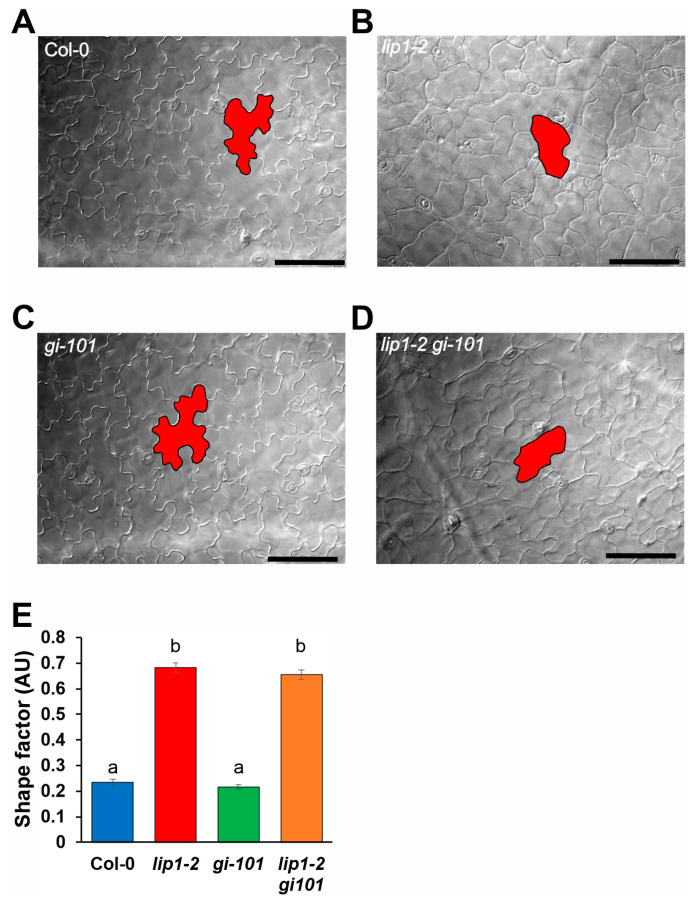
The pavement cell morphology phenotype of *lip1-2* mutants is independent of GI. Pavement cell morphology of Col-0 (**A**), *lip1-2* (**B**), *gi-101* (**C**) and *lip1-2 gi-101* (**D**) plants grown in 12 h light: 12 h dark photocycles for 8 days. Representative cells are outlined with black and filled with red colours to aid visualisation. Scale bars: 100 µm. (**E**) Cell shape factor values were calculated from the area and the perimeter of cotyledon pavement cells. n = 34−45, Error bars indicate standard error, and different letters show significant differences at *p* < 0.01 (Duncan’s test).

Previously, we described that epidermal pavement cells of *lip1-1* and *lip1-2* mutants show rounded shapes, probably due to increased ploidy levels, which is the result of impaired suppression of endoreplication [24]. We monitored this phenotype as a proxy for ploidy levels in the different genetic backgrounds. Figure 7 illustrates that, in agreement with previous results, the shape of pavement cells of the cotyledons in 7-day-old light-grown *lip1-2* seedlings (Figure 7B) was much less complex compared to the wild-type plants (Figure 7A). The *gi-101* mutant did not show obvious alterations from the wild type (Figure 7C). The *lip1-2 gi-101* double mutant (Figure 7D) phenocopied the *lip1-2* single, indicating that GI is not required for the manifestation of the cell shape phenotype of *lip1-2*. These conclusions were fully supported by calculating the shape factor, which describes the roundness of cotyledon pavement cells of the four genotypes tested (Figure 7E). Values for *lip1-2* and *lip1-2 gi-101* were identical and significantly different from that of the wild type and *gi-101*. These results also demonstrate that loss of GI function does not alter cell morphology.

**Figure 8:**
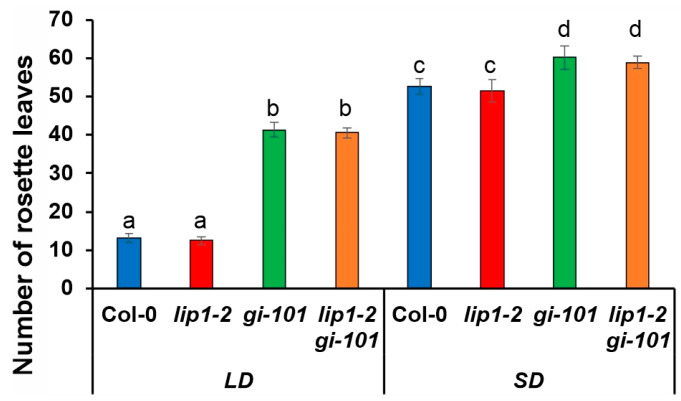
LIP1 does not affect photoperiodic flowering. Col-0, *lip1-2*, *gi-101* and *lip1-2 gi-101* plants were grown in soil in 16 h light/8 h dark (LD) or 8 h light: 16 h dark (SD) photocycles at 22 °C. Rosette leaves were counted when inflorescences reached 1 cm. A total of 12–15 plants were analysed for each genotype and condition. Error bars indicate standard error, and different letters show significant differences at *p* < 0.01 (Duncan’s test).

GI is a key player of photoperiodic flowering upregulating *FT* transcription by CO-dependent [20] and CO-independent [41] routes. Accordingly, flowering is dramatically delayed in loss-of-function *gi* mutants. Owing to the lack of information on the flowering phenotype of *lip1-2* mutants, plants of the different genotypes were grown in short-day (8 h light/16 h dark, SD) or long-day (16 h light/8 h dark, LD) conditions. The time of flowering was determined as the number of rosette leaves at bolting. Figure 8 demonstrates that flowering time was not altered in the *lip1-2* mutant in either condition. The *gi-101* single flowered later than the wild type, whereas the *lip1-2 gi-101* double was indistinguishable from the *gi-101* single.

These results demonstrate that the function of GI in flowering time initiation is not modulated by LIP1.

## 4. Discussion

LIP1 is the first small GTPase that has been functionally linked to the circadian clock in plants. Lack of LIP1 function results in an accelerated circadian oscillator producing short period rhythms. However, the mechanism by which LIP1 affects the oscillator remained unknown. In the present work, we aimed at revealing an essential piece of this regulation and identify the particular oscillator component that is primarily targeted by LIP1. 

We tested the mRNA accumulation of several key clock genes in the loss-of-function allele *lip1-2* in continuous low fluence red light, where the short-period phenotype is most pronounced. No significant changes in mRNA levels of clock genes were found, suggesting that transcriptional modulation is not the principal effect of LIP1 on the clock. The pace of the oscillator can also be influenced by altering the function or the turnover of one or more clock proteins. This effect may be mediated via protein–protein interactions. Although direct interaction between LIP1 and clock proteins was not detected, a search for binding partners identified a guanine exchange factor (GEF7), which in turn showed physical interaction with GI, TOC1 and ZTL proteins. GEF7 is a member of the RopGEF protein family [32] and acts as a functional guanine exchange factor to activate Rop GTPase AtRAC1, required for root meristem maintenance [42]. GEF7 was also shown to interact with the eukaryotic translation initiation factor 4E1 (eIF4E1), indicating that the function of eIF4E1 in the regulation of auxin-dependent embryo development and primary root initiation may partially depend on ROP signalling [43]. Interestingly, pairwise interactions between GI, TOC1 and ZTL were also found in Y2H assays. Supposing that these interactions take place in vivo, the results suggest the existence of a multiprotein complex to which LIP1 may bind through GEF7. It is notable that these factors, including LIP1, operate in the evening [23]. To link LIP1 to the oscillator, one can assume that the complex of GI-TOC1-ZTL could modulate the activity of LIP1 through GEF7, and then LIP1 could affect the function of one or more clock proteins. Alternatively, LIP1, brought into proximity by GEF7, could influence the activity of GI, or TOC1, or ZTL. The analysis of epistasis between LIP1 and the three clock components supported the latter option and identified GI as the downstream target of LIP1-derived signalling to the oscillator. 

LIP1 was first identified by the circadian phenotype of *lip1-1* mutants [23], but several other pleiotropic functions have been described since [24]. We showed that LIP1 affects light-controlled hypocotyl elongation, endoreduplication and salt-stress responses [24]. GI has even more diverse functions in regulating a wide range of physiological processes [17,18], which partially overlap with those affected by LIP1. Thus, it was a reasonable hypothesis that maybe several or all pleiotropic functions of LIP1 are exerted via the modulation of GI activity. However, an analysis of *lip1-2 gi-101* double mutant plants revealed that none of the other functions of LIP1 require GI. The clear additivity of quantitative phenotypes indicated that photomorphogenic traits and salt-stress responses are regulated by both LIP1 and GI, but via different mechanisms. On the other hand, the regulation of cell morphology is a function not shared by LIP1 and GI, since the loss of GI function did not alter the shape of pavement cells either in the wild type or the *lip1-2* mutant backgrounds.

How does LIP1 modulate the activity of GI? A simple way of control could be the regulation of the level/turnover or the subcellular localization of the GI protein. However, these changes should alter all functions of GI, including flowering time determination [44,45], which phenotype was not observed in *lip1-2* mutants. This indicates that LIP1 does not affect the function of GI in general, but probably acts selectively on a particular branch of GI signalling. The distinct pleiotropic functions of GI appear to be realized via specific protein–protein interactions. Based on our current results, and considering published data, we hypothesize that LIP1 affects the interaction of GI with only those downstream effector proteins that relay the effect of GI on the clock. In contrast, interactions representing outputs of GI towards the regulation of salt stress, photomorphogenesis and flowering should not be affected by LIP1. GI regulates the circadian oscillator via at least two distinct mechanisms. GI triggers the destabilization of PIF transcription factors, and thus relieves repression on *CCA1* transcription [46]. On the other hand, GI stabilizes ZTL in the light, which in turn mediates the degradation of clock proteins, among them TOC1, in the dark [47]. Since *CCA1* mRNA levels were not altered in *lip1-2,* and *ztl-3* was not epistatic to *lip1-2*, we concluded that these two regulatory mechanisms of GI are probably not influenced by LIP1. Rather, these data indicate the existence of an additional, LIP1-modulated functional link from GI to the clock.

**Figure 9:**
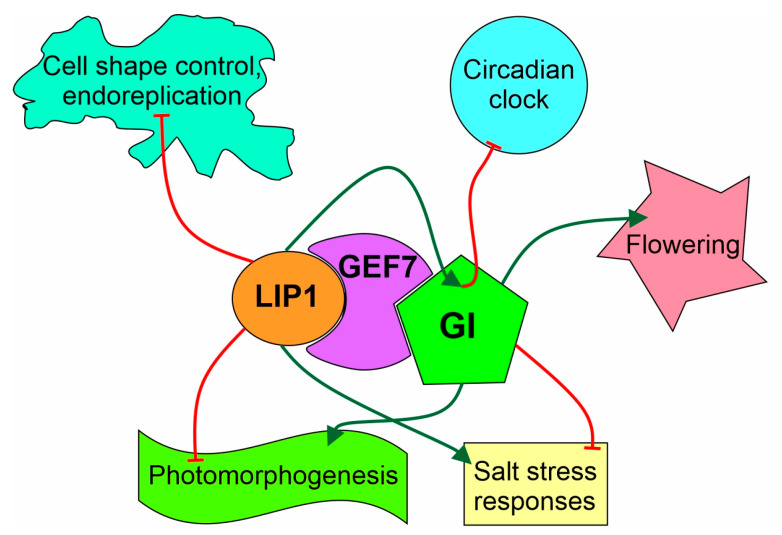
Model illustrating protein level and functional interactions of LIP1 and GI. LIP1 indirectly binds to GI via GEF7 and regulates the circadian clock by enhancing the clock-specific function of GI. LIP1 and GI have opposite effects on salt-stress responses and photomorphogenesis through independent signalling routes. LIP1 attenuates endoreplication and GI promotes flowering, but these two regulatory functions are not shared by LIP1 and GI. Green arrows or red blunt-ended lines indicate positive or negative effects, respectively.

Loss-of-function mutations in LIP1 result in accelerated circadian oscillations with periods shorter than that of the wild types [23]. GI is a unique component of the oscillator, since both the reduction (e.g., in *gi* mutants) and the increase (e.g., GI overexpressing plants, GI-OX) in GI activity results in short-period rhythms [44,46]. However, the period phenotype in GI-OX is accompanied by a phase delay, which cannot be detected in rhythmic traces from *gi* mutants. Considering these characteristics, the circadian phenotype of *lip1-2* is more similar to that of the *gi* mutants, and not the GI-OX plants. Thus, we propose that LIP1 positively modulates the circadian function of GI (Figure 9). 

In summary, we demonstrated that (i) LIP1 affects the circadian clock via selectively affecting a specific function of GI and (ii) regulates salt stress and photomorphogenic responses by GI-independent routes, (iii) but does not affect the photoperiodic pathway of flowering initiation.

## Figures and Tables

**Table 1:** Period estimates demonstrate genetic interaction between LIP1 and GI.

Marker	Genotype	Period (h) ± SE	*p*-Values
**CCR2:LUC**	Col	27.71 ± 0.25	<0.001
	*lip1-2*	24.41 ± 0.29	0.009
	***gi-101***	**25.91 ± 0.23**	**0.35**
	***lip1-2** **gi-101***	**25.72 ± 0.51**	**-**
**CAB2:LUC**	Col	27.36 ± 0.52	<0.001
	*lip1-2*	25.02 ± 0.27	0.016
	*toc1-4*	25.88 ± 0.38	0.006
	*lip1-2 toc1-4*	24.26 ± 0.41	-
**CCR2:LUC**	Col	27.81 ± 0.33	0.002
	*lip1-2*	24.16 ± 0.71	<0.001
	*ztl-3*	31.12 ± 0.44	0.021
	*lip1-2 ztl-3*	29.48 ± 0.63	-
**CCR2:LUC**	Col	27.43 ± 0.38	<0.001
	*lip1-2*	24.38 ± 0.54	0.027
	*cca1-1*	25.79 ± 0.65	0.007
	*lip1-2 cca1-1*	23.51 ± 0.18	-

Luminescence data of plants shown in Figure 3 were analysed by the BRASS2 software package. Free-running periods were estimated by FFT-NLLS analysis. Rhythmic traces from all independent experiments were analysed and averaged. *n* = 72–96 per genotype. Variance-weighted period and standard error (SE) values are shown. *p*-values were calculated from pairwise *t*-tests to determine the significance of differences from the corresponding double mutant in terms of periods.

## Data Availability

The data supporting the findings of this study are available within the paper and Supplementary Materials.

## Supplementary Materials

The following supporting information can be downloaded at: https://www.mdpi.com/article/10.3390/cells13171503/s1, Table S1: List and sequences of oligonucleotides used for molecular cloning, genotyping or qPCR assays.
