# Supplementary material for: LIP1 Regulates the Plant Circadian Oscillator by Modulating the Function of the Clock Component GIGANTEA
Source: Cells. 2024 Sep 8;13(17):1503. doi: 10.3390/cells13171503 (PMC11394198; doi:10.3390/cells13171503)
Supplement: Supplementary file 1 [file cells-13-01503-s001.zip › cells-3149823-supplementary.pdf]

Table S1. List and sequences of oligonucleotides used for qPCR assays, molecular cloning or genotyping.

Name or ID	Sequence (5'->3')	Purpose
TOC1 RT Fwd	ATCTTCGCAGAATCCCTGTGATA	qPCR primers used for quantitative analysis of mRNA levels.
TOC1 RT Rev	GCACCTAGCTTCAAGCACTTTACA	
CCA1 RT Fwd	CTGTGTCTGACGAGGGTCGAA	
CCA1 RT Rev	ATATGTAAAACTTTGCGGCAATACCT	
GI RT Fwd	AATTCAGCACGCGCCTATTG	
GI RT Rev	GTTGCTTCTGCTGCAGGAACTT	
PRR5 RT Fwd	GTGTATGTTGAAAGGTGCGG	
PRR5 RT Rev	AGGAGCAAGTGAAGTTTGTC	
PRR7 RT Fwd	GTAGAAACTGTGATCTGGCCCTG	
PRR7 RT Rev	GCACATTCCGATCATCCCTAA	
PRR9 RT Fwd	GCCTTCTCAAGATTTGAGGAAAGC	
PRR9 RT Rev	TTTGGCTCACCTGAAGTACTCTC	
ELF4 RT Fwd	CGACAATCACCAATCGAGAATG	
ELF4 RT Rev	AATGTTTCCGTTGAGTTCTTGAATC	
LUX RT Fwd	GACGATGATTCTGATGATAAGG	
LUX RT Rev	CAGTTTATGCACATCATATGGG	
TUB2/3 RT Fwd	CCAGCTTTGGTGATTTGAAC	
TUB2/3 RT Fwd	CAAGCTTTCGGAGGTCAGAG	
LIP1 CDS F	TCGCGAATTCATGAAGTTTTGGAGGGAACGTGAAAG	PCR-amplification and cloning of coding regions of the genes listed in pGBKT7 and pGADT7 vectors used for yeast two-hybrid assays.
LIP1 CDS R	TCGGGGATCCTCAGACGTTAATATCCATTCGCTTTGAC	
GEF7 CDS F	TCGGGATCCTTATGGATGGTTCGTCGG	
GEF7 CDS R	TATGAGCTCTCAAATCCCAGGATCAAGG	
GI CDS F	CTTTTGCGAATTCATGGCTAGTTCATCTTCATCTGAGAGA	
GI CDS R	TTTGCGCTCGAGTTAGCGGCCGCATTGGGACAAGGATATAGT	
TOC1 CDS F	TTGGCTCGAGGAATTCATGGATTTGAACGGTGAGTGTAAAGG	
TOC1 CDS R	TTCTGAGCTCCTACTCGAGAGTTCCCAAAGCATCATCCTG	
ZTL CDS F	TGGACTCGAGGGATCCGTATGGAGTGGGACAGTGGTTC	
ZTL CDS R	TTTCCCGGGTTACTCGAGATTCGTGAGATAGCTCGCTAGTGAT	
gi-101 LP	GATCGATGGTCTTCAGTTCTCTTC	Primers used for PCR-genotyping the gi-101 mutant. LP+RP recognises the WT allele, BP+RP recognises the T-DNA insertion allele of GI.
gi-101 RP	GCTGTTTCATCAGAAAACGAAA	
gi-101 BP (LBB1.3)	ATTTTGCCGATTTCGGAAC	
